# Our Summer at Maplewood. Chapter XI, Little Things

**Published:** 1875-12

**Authors:** 


### CHAPTER XI.

### LITTLE THINGS.

"Cousin Kate, are you going to ignore cake entirely in your cook-book?" asked Alice, one evening at tea, as she passed me the loaf-cake she had made a week before. "Even you sometimes indulge in eating cake."

"Very true, Alice," I replied, "I enjoy this bread-cake, for instance; and quite delight in good ginger-cake at times. But I am so disgusted with the prominence given in cook-books to cake over bread, and I everywhere see so much time and attention bestowed upon cake, and so little upon bread, that I am strongly tempted, in my cook-book, to treat cake of all kinds with silent contempt."

"But," said Emmeline, "since people will eat cake why not tell them how to make a few varieties that are inexpensive and comparatively harmless? This loaf or bread-cake is to my taste better than the average fruit-cake, for it has a cleaner taste. Yet it dosn't cost quarter as much as the fruit cake, and is easier made—isn't it Alice?"

"Yes, mamma, this cake gave me no more trouble to make, than would a loaf of bread. From the bread when perfectly light and ready for the last moulding, I took three cups of dough, to which I added two cups of white sugar," one cup of butter, two eggs well beaten, one cup chopped raisins one teaspoonful cinnamon, and half a teaspoonful of soda. As the dough was very high I pressed it into the cup, measuring it as full as possible. Placing these ingredients together in an earthen bowl, I worked them with my hand for half an hour. The mixture became quite soft after being worked awhile. I put it into a well buttered baking dish, lined with white paper, and, placing it upon the table covered, let it stand two hours to rise. When light—which was shown by little bubbles of air dotting its surface, more than from its having risen—I placed it in a very moderate oven, not nearly so hot as bread requires, and every few minutes I opened the oven door just enough to peep in and see how it was baking. To my joyful surprise—for it being my first attempt at bread-cake, I scarcely looked for success,—each time I peeped, I found it rising, rising, rising, slowly, and at last I shut the door, satisfied I could leave it to bake unwatched. It was in the oven an hour and a half, and you see it came out very nice."

"And how well it keeps!" observed Emmeline; "Indeed, I think age improves it, as it does many things."

"And people, also," I answered. "Did it ever occur to you, Emmeline, how many men and women ripen and mellow—growing sweeter and more tender as the autumn of life comes on? This break-cake," I continued, "to return to the subject, when baked in suitable form, makes a very nice pudding, if steamed, and served hot, with wine or fruit-sauce. To my taste it is more palatable than the ordinary plum pudding, while it is much less indigestible. Bread-cake doughnuts and buns—"

"No buns for me," said Emmeline.

"I detest the whole rusk family. Many people like that half and half sort of food—a something between one thing and another—a link joining this to that, but my taste is different. If I eat bread, I want it to be bread; if cake, cake; not a combination of both, resulting in neither."

"But, mamma," said Alice, "buns" are bread etheralized—light, airy things that I delight in eating because of their very daintiness. Cousin Kate, my recipe for buns reads in this manner: Whip together three ounces of sugar and three eggs, until well broken and mixed, then pour upon them slowly, continuing the beating meanwhile, a pint of boiling milk. Add a pint of flour, and when lukewarm a half gill of yeast. Beat all well together, cover closely, and let stand over night. In the morning add a handful of flour, and beat them soundly. When again light, work well with the hand, adding three ounces of butter, and flour very gradually. The excellence of these buns consists mainly in their being soft and elastic or spongy. When the dough is stiff enough to cling together and work away from the sides of the bowl, sufficient flour has been added. Work ten or fifteen minutes longer, cover closely, and leave to rise. When very light mould into small round cakes, and place in a pan, not allowing the cakes to touch each other. Let them rise in the pan until light, then bake in a moderate oven. Is that right, cousin Kate?"

"You have omitted flavoring and citron," I said.

"So I have," answered Alice, "I forgot to mention that at the time of adding the butter, lemon and other flavoring essences be used according to taste; and that a slice of citron, placed in each bun at the time of forming, gives them a nice flavor, and causes a delightful surprise to the uninitiated eater. By the way, how will this do for doughnuts? Put into a pan three pints of sifted flour. Make a well in the centre, into which put a half pound of sugar, a gill of buttermilk or thick sour milk, two eggs, two ounces of butter, a tea-spoonful of soda, and flavoring to taste. With the hand mix these ingredients well together, working in the flour gradually, until the dough is very smooth. It must not, however, be made stiff. Roll upon the moulding board half an inch thick, cut in form, and fry in boiling lard. When cool, dust the doughnuts with pulverized sugar. Doughnuts made according to this recipe are the richest that I ever ate."

"The next time you make buns, Alice, when the dough is ready to shape, roll it on the moulding-board, cut into doughnuts and fry in boiling lard. And you will, I think, decidedly give them the preference over those made according to your recipe."

"When the flavoring is omitted, and the doughnuts are dusted with pulverized sugar and cinnamon, they suit my taste much better," said Emmeline. "And Kate," she added, "a good doughnut isn't a bad thing with a cup of coffee for breakfast."

"A doughnut and a cup of coffee," I answered, "may be well enough for breakfast, sometimes. But, in my opinion, a nice roll, or a slice of good bread, with sweet rich butter, far outranks doughnuts or cake as an accompaniment of coffee."

"Kate, what sort of pickles do you like best, with fried or scolloped oysters?" asked Emmeline, turning suddenly from contemplation of sweet things to things sour.

"Cold-slaw," I replied; "cabbage crisp and sweet, with a simple salad dressing. I like it a thousand times better than any pickle that ever was made. Next to eating such quantities of cake and pastry, I think Americans are most absurd in their free use of pickles and condiments. When people can have sliced tomatoes, fresh and delicious, or cool, crisp cabbage, or sour baked apples full of tart spiciness, it is a marvel to me that they should crave mustard, catsup, and pickles. Pickles may be useful, and entitled to a place on our tables in the late winter, or early spring, when fresh vegetables and acid fruits are hard to obtain, and I shall give a few receipts for making them; for it is a pity that house-wives should buy, at an exorbitant price, what, with a little trouble, they can make much better for themselves. First, among pickles, I shall give chopped cabbage. Select cabbage crisp and sweet; chop it moderately fine; season to taste with white mustard-seed, salt and pepper. Put it in a jar and cover with cold vinegar. Scatter whole cloves over the top, to prevent mold. Do you know, Emmeline, it is said that cloves scattered over any pickle, sweet or sour, will effectually prevent mold? and, so far as I have tested, I have found it to be true. If preferred, sweet peppers may be chopped with the cabbage, instead of using ground pepper."

"It occurs to me, Cousin Kate," said Alice, "this seems a very loose sort of a receipe for you to give who are so particular about weights and measures. You say, 'season to taste.' I think you should designate the amount of salt and pepper."

"Not at all," I replied. "Spicing and flavoring are matters of taste. Some like much, others less; and I think every housewife should use her own discretion, and decide for herself about such matters. Besides, Alice, what I desire above all things is to induce women to think and experiment for themselves in the matter of cooking—to seek out many new ways and inventions, so as to secure improvements upon the old methods. Just as soon as women become convinced that this question of how best to prepare the food we eat, is a momentous one, and that she who excels in answering it deserves high rank among women—and not only among women, but among public benefactors,—good cooks will abound."

"But when will they become convinced?" asked Emmeline, with a sigh.

"Not so long, at least," I answered, "as mothers allow their tables to be furnished with hotly spiced chow-chow and other such abominations, when it is so easy, by taking a little trouble, to substitute this mild tomato-sauce:—Take twelve ripe tomatoes; peel and slice them. Chop fine, four sweet peppers, ripe or green, and two onions. Place all together in a preserving kettle, adding two table-spoonfulls of salt, two of sugar, and a pint of vinegar. Simmer two hours, or until quite thick; then bottle for use. Or this green tomato sauce :—Take a peck of green tomatoes and ten onions; slice and sprinkle lightly with salt : Let it stand over night. In the morning drain the brine or water away from it, and put in a preserving kettle with three half gallons of vinegar, three sweet peppers, chopped fine; a quarter of a pound of white mustard-seed, and an ounce of cloves. Boil slowly three or four hours, stirring frequently. Half an hour before removing from the fire add one pound of sugar. Or this cucumber-catsup which is simple and easily made, yet full of pungency. Grate six green cucumbers and three onions. Add to the grated mixture a teaspoonful of salt, and half a teaspoonful of pepper. Drain off the juice, and, after measuring, throw it away, adding to the catsup the same quantity of vinegar. Mix well and bottle for use."

"But," said Emmeline, many people prefer cucumber pickles, pure and simple, to all mixtures, and fancy preparations. Do you know the best method of making them?"

"Perhaps not. But this is a very excellent method : Put the freshly gathered cucumbers in a jar, sprinkle lightly with salt, and when the jar is full of cucumbers pour on as much boiling water as it will hold. After the water becomes cold, remove the cucumbers to a preserving kettle, lined with green grape leaves. Add a small piece of alum, if you wish to improve the color by the addition of an unwholesome ingredient, cover with weak vinegar, and heat to boiling point. Remove, and drain the pickles, and put them in a jar, distributing among them pieces of sweet pepper. Cover with strong vinegar, in which a few cloves and a small quantity of sugar have been heated."

"How many cloves and how much sugar?" asked Alice.

"An ounce of cloves and a pound of sugar to a gallon of vinegar, is what *I* should use," I replied; "but that is a matter of taste, and, as I said before, I prefer, in such cases not to give weights or measures. I will also add, Alice, that when I speak of vinegar I always mean good, strong vinegar, and not the weak, watery, insipid stuff that is often used by housewives, with the almost certain loss of the pickles on which it is used. Yet I notice that people whose pickles spoil from want of good vinegar, generally attribute their having spoiled to some other cause."

"Cousin Kate, I hope you will give some nice methods of sweet pickling, or spicing fruits. Among pickles, sweet or spiced ones are my favorites, although mamma, no doubt, would object to them as occupying neutral ground between pickles and preserves, as being too undecided, not positive enough, in character to suit her."

"Spiced peaches and damsons are both very nice," I answered. The former I prepare in this manner:

Selecting hard, free-stone peaches, I cut in halves, and remove the stone before paring, as I find it much easier to remove before, than after paring. For seven pounds of fruit I use three pounds of sugar, a quart of vinegar, and a half ounce each of cloves and cinnamon. I put the sugar with three pints of water into a preserving kettle, and when it boils, cook as many of the peaches at a time, as the kettle will hold without their being piled one upon another· I cook slowly, turning them over and shaking them about, that they may cook evenly; and when tender, remove by means of a wire skimmer and place in a jar. When all the fruit is cooked, I simmer the syrup until quite thick, then add vinegar and spice, simmer altogether a few minutes, and pour over the peaches.

Damsons I treat differently. Selecting large fair plums, I puncture the skins with a fork, and lay them in a stone jar. Then I heat to boiling four pounds of sugar and a quart of vinegar, with spice, the same as for peaches. When boiling hot I pour the liquor upon the plums, cover closely and set aside until next morning; I drain from the plums all of the juice, heat to boiling, and pour over them as at first. I repeat this every morning for a week, or until the damsons are soft and tender. I then put all in the kettle together, simmer gently for a few minutes, skim out the fruit and put it in a jar; simmer the syrup twenty or thirty minutes, or until it seems rich and sufficiently thick, then pour it over the fruit, and consider the process of spicing damsons completed. But here comes Tom with our letters and papers.

Our trunks were packed and our arrangements all completed for leaveing Maplewood; and as we sat on the piazza in the September twilight, listening to the chirping of the Katy-Did, Alice observed.

"Cousin Kate I would like to linger on here indefinitely. I have never passed a pleasanter summer; and I wish we could stay through October and November, at least to watch the Autum foilage as it brightens and fades.

"If I had known before about making other arrangements" replied Emeline, "that Mrs Douglass had concluded to remain abroad, I could have have been satisfied to remain several months longer; for the summer here has been to me very restful. True, I have not made much progress with my novel. But I think my time has been spent to good advantage. I have looked at life from a different stand-point than before and begin to feel a deep interest in every thing that concerns our common humanity. I know I do not realize, as Kate does, its needs and its wants, but I accept the fact that no true woman ought to remain idle or take to novel-writing merely for amusement or to kill time. The great majority of men and women know so imperfectly how to do the little every-day duties of life, the aggregate of which makes up the sum of human happiness, that they go stumbling and blundering through the world hoping that somehow, or somewhere in the far off future, they will come up with and enjoy the good time, which through ignorance and laziness they fail to grasp, and make for themselves here."

"Ma," interrupted Alice, "you accused me of being unorthodox when I told you Gerald's musings were such fancies as I frequently indulge in myself, and were not 'wild ravings' as you term them. It seems to me *you* are stepping off the orthodox platform when you condemn people for hoping and expecting to have a good time prepared for them by some unseen power, when they are too stupid or lazy to make it for themselves here, where they have the materials for so doing lying all about them."

"Alice," I interposed, "you and your mother seem to me to differ about non-essentials only. You both admit that your views of life have changed within the past few months; and you both agree that an idle, selfish aimless life is the lowest possible form of human existence. It is very evident therefore, that you have both changed positions: and if by orthodoxy you mean remaining stationary—always of the same belief—it must be apparent to both of you that you are not so orthodox as you were a few months ago. People who use their eyes and brains learn to see and do things differently, and of course to think differently about them, nearly every day. And until they do use these organs, it is folly for us to expect much progress or improvement to be made in the world. The great majority of people, as you say, do not know how to do the little every day duties of life that lie all about them. Why do they not? Is it from ignorance, stupidity or laziness? It seems scarcely credible that men and women with ordinary sense can go through the world without noticing the thousands of little things that are component parts of their every day life. It is hard to believe that a girl could grow up in a house where three meals are prepared every day, and when she arrives at womanhood have no knowledge of how to prepare a breakfast dinner or supper. Yet I presume each of us is personally acquainted with fifty young women who do not know how to broil a beefsteak, or bake a potato; and haven't the faintest idea of the first principles of bread-making. It is a burning shame to our sex that so many of us are content to grow up thus ignorant and no wonder women have not obtained the ballot. But while men sneer at us, and with justice too, for such shiftlessness and inefficency, they are, in respect to using their eyes and brains, not one whit better that women. I know scores of great, hulking fellows who have warmed themselves by coal fires all their lives, and yet can't kindle one in a grate or stove; or can't, when it is kindled, keep it burning two consecutive days. It is the rule, and not the exception, to forget and neglect life's every day duties. The world suffers more from heedlessness than it does from criminality; and we wrong ourselves and others as much by want of thought, as we do by want of heart. We are continually worrying and annoying ourselves and all who are intimately associated with us by neglecting, or forgetting to do our every day duties. Our eyes and brains were given us for a beneficent purpose ; but most of us fail to discover their use or to use them to much practical advantage. A great portion of life is wasted and much vital force consumed in corecting the evil results of ignorance, negligence and forgetfulness; or in doing labor that could be avoided by a moderate use of common sense. Why should any house-wife suffer her fires to go out every few days in mid-winter through carelessness, and thus endanger the health of some member of the family, as well as waste the time of some one in rebuilding them ? Or, why should she induce dispepsia, discomfort, and general disgust in the household, by making bad bread, bad butter, or bad food of any kind when she could have the best of everything by a little care and attention? Or why should men and women who have homes permit things in and about them, to get out of order and remain so for weeks and months, to the almost hourly annoyance and discomfort of every one in the family? Go among the homes all over our land, and in a majority of them you will find the occupants suffering discomfort and inconvenience from a dozen trifling things that they do not seem to observe; or, are too stupid and inefficient to remedy. For instance, a door sags on account of a loose screw in one of its hinges, and causes extra labor every time it is opened or closed; or, its hinges squeak for need of a few drops of oil, and disturb every one in the house a dozen times a day ; or a window shutter bangs and flaps for want of a fastening, till it eventually tumbles off and is broken; or a pump gets out of order, and for want of a slight repair, has to be labored with everytime a bucket of water is needed. And so I could go on and on and mention a thousand similiar things that any one of thought and observation can see almost daily, and on every one of which extra labor and strength are wasted, week after week, and month after month, when a few moments thoughtful, intelligent effort would put any one of them in perfect condition. Yet people endure such extra labor and discomfort without seeming to realize that they can avoid it by removing the cause. Through all the various departments of society the disposition to forget, neglect, overlook, or remain ignorant how to do minor matters, as they are termed, is so apparent that it is rather difficult to get a correct idea of what the average man and woman conceive to be the chief end of existence. I am old fashioned enough to think that people who endeavor to escape the so-called drudgeries of life, by shirking and neglecting its ever present duties and responsibilities, soon become incapable of appreciating and enjoying its purest delights and satisfactions, and fritter away their time, vainly searching for a sphere in which they can labor to the best advantage ; or spend it, avariciously striving to get up " a corner " on the fruits of the Future, and monopolize the whole stock of happiness supposed to be garnered up in the storehouse of the " good time coming."

"Kate, is seems to me you are right in the main," said Emmeline. "But you wouldn't have people give too much time to little things would you?"

"Too much time? Why, Emmeline, the more I think upon this subject the more I am impressed with its magnitude and importance. What do people mean when they talk of being so engrossed with great things that they forget or neglect, little ones? Great and small are but relative terms; and the great thing is simply the aggregate of many small ones. Who can determine where the dividing line between big and little things should be drawn; or exactly how many little things are required in the composition of one big thing? Is the size of things, their importance or unimportance, their bigness or littleness accurately computed by the amount of time, labor, care, strength or intellect bestowed upon them; or by the effect they produce upon the world? Or is it carelessly measured by an imaginary standard for whose correctness no one is responsible? Are the great things of life those that give society at large the greatest amount of happiness; or those that yield a few persons the greatest amount of money? Does the surgeon who performs a dangerous operation on a patient do a great thing, and the nurse who faithfully watches and ministers to that patient, do a small thing? Does the preacher who delivers a learned doctrinal discourse do a great thing, and the layman who by a few kind words encourages some weak or wayward brother or sister to live a purer and better life, do a little thing? Does the educated lawyer, who, for a heavy fee, defends and acquits a criminal, do a great thing, and the uneducated boor who, without pay, protects the property, or saves the life of a neighbor do a little thing? Does the stock-jobber who amasses a million in a month by obtaining control of a corporation, and by watering or skillfully manipulating its stock do a big thing, and the mechanic who toils faithfully a life-time and earns only his daily bread do a little thing? Does the speculator who buys up the products of a thousand farmers or work-shops, and by thus controlling the market, makes a fortune in a week, do a big thing; while the owners of those farms and workshops who toiled diligently for months, and obtained but small gains, did little things? Does the man of wealth who buys a tract of land for a few thousand dollars, and holds it unimproved till it increases in value to millions, do a big thing; and do the thousands of men of humble means who buy of him at the increased price, and improve and beautify their purchases with pleasant homes· do little things? Who shall say that the woman remains quietly at home doing daily and with systematic neatness, the housewifely duties that render all within her influence contented and happy, is not teaching her sister women as wisely, and doing as much for the world's moral, social, and political advancement, as the one who travels from the Atlantic to the Pacific, eloquently pleading the right of her sex to larger liberty or wider priveleges? Or, which one of us is able to decide that a little poem wrought out in obscurity, and for which its author received a few dollars only, has not effected a greater good in the world than the gigantic enterprise inaugurated with ostentatious display and applause, that has put millions in the pocket of its projector?

If then we accept the principle that it is the duty of every man and woman to be engaged in work that is useful in some way to the world, why is it not as well to make beds, sweep rooms, and cook dinners, as it is to plough fields, build houses, and buy and sell corn and cotton? And when a women's duty lies in the way of the former, rather than the latter occupations, why should it be distasteful to her to acquaint herself with all the details of housekeeping? Or, why should she consider it drudgery to spend her time in doing, in the most perfect manner, those little household economies that add so much to the comfort and happiness of life?

To teach their children self-restraint—the control of their appetites and passions—should be a paramount consideration with all parents; for people who simply live to eat, or as the poet puts it:

> "——————who creep
> Into the world to eat and sleep,
> And know no reason why they're born
> But merely to consume the corn,
> Devour the cattle, fowl and fish,
> And leave behind an empty dish,"—

are vagabonds and drones, who steal and consume the substance of those who labor and produce. Nevertheless, we must all eat to live; and the food we eat should be prepared in the best manner. Men and women, are the most perfect creations of the Maker of the universe, and are designed for the performance of labor of the highest order; but unless the conditions of their existence are complied with, and the materials that are required to keep them in working order are prepared with skillfulness and care, it is impossible for them to do the best of work and lead the truest of lives.

The sum of the whole matter then, is this: That the great things of life are composed of, and dependant on, its little things; and the more care and attention that are bestowed upon necessary little things the more successful will be the endeavor, and the greater the result, of all human effort."

"Cousin Kate," said Alice, "as you commenced your discourse without a text you might appropriately terminate it with one. For instance,

> "Little drops of water
> Little grains of sand,
> Make the mighty ocean
> And the pleasant land."

And since you appear to be able to preach almost as well as you can cook, you might, in case you relinquish your idea of publishing a cook-book, favor the world with a volume of sermons. But jesting aside, I think I have learned immeasurably more this summer than ever before in the same length of time: and I am sure I have never been more contented and happy."

"Alice you express my sentiments also," said Emmeline. "Therefore if my novel should remain forever unfinished, and cousin Kate's cook-book should never be written: even should our expectations fail to be realized in the Home-School—still the lives of us three will be richer and sweeter, for having spent OUR SUMMER AT MAPLEWOOD.

THE END.

